# The importance of stroke as a risk factor of cognitive decline in community dwelling older and oldest peoples: the SONIC study

**DOI:** 10.1186/s12877-020-1423-5

**Published:** 2020-01-22

**Authors:** Werayuth Srithumsuk, Mai Kabayama, Yasuyuki Gondo, Yukie Masui, Yuya Akagi, Nonglak Klinpudtan, Eri Kiyoshige, Kayo Godai, Ken Sugimoto, Hiroshi Akasaka, Yoichi Takami, Yasushi Takeya, Koichi Yamamoto, Kazunori Ikebe, Madoka Ogawa, Hiroki Inagaki, Tatsuro Ishizaki, Yasumichi Arai, Hiromi Rakugi, Kei Kamide

**Affiliations:** 1 Department of Health Promotion System Sciences, Division of Health Sciences, Graduate School of Medicine, Osaka University, Osaka, Japan; 2 Department of Clinical Thanatology and Geriatric Behavioral Science, Graduate School of Human Sciences, Osaka University, Osaka, Japan; 3 Tokyo Metropolitan Geriatric Hospital and Institute of Gerontology, Tokyo, Japan; 4 Department of Geriatric and General Medicine, Graduate School of Medicine, Osaka University, Osaka, Japan; 5 Department of Prosthodontics, Gerodontology and Oral Rehabilitation, Graduate School of Dentistry, Osaka University, Osaka, Japan; 6 Center for Supercentenarian Medical Research, Keio University School of Medicine, Tokyo, Japan

**Keywords:** Stroke, Cognitive decline, Older and oldest people

## Abstract

**Background:**

Cognitive impairment is a major health concern among older and oldest people. Moreover, stroke is a relevant contributor for cognitive decline and development of dementia. The study of cognitive decline focused on stroke as the important risk factor by recruiting older and oldest is still lagging behind. Therefore, the aim of this study was to investigate the importance of stroke as a risk factor of cognitive decline during 3 years in community dwelling older and oldest people.

**Methods:**

This study was longitudinal study with a 3-year follow-up in Japan. The participants were 1333 community dwelling older and oldest people (70 years old = 675, 80 years old = 589, and 90 years old = 69). Data collected included basic data (age, sex, and history of stroke), vascular risk factors (hypertension, diabetes mellitus, dyslipidemia, atrial fibrillation, and current smoking), and social factors (educational level, frequency of going outdoors, long-term care (LTC) service used, and residential area). The Japanese version of the Montreal Cognitive Assessment (MoCA-J) was decline of ≥2 points was defined as cognitive decline. Multiple logistic regression analysis was used to investigate the association between stroke and other risk factors with cognitive decline during a 3-year follow-up.

**Results:**

The fit of the hypothesized model by multiple logistic regression showed that a history of stroke, advanced age, and greater MoCA-J score at the baseline were important risk factors, while the presence of dyslipidemia and a higher educational level were protective factors that were significantly correlated with cognitive decline during the 3-year follow-up.

**Conclusions:**

The cognitive decline after the 3-year follow-up was influenced by the history of stroke and advanced age, while greater MoCA-J score at the baseline was positively associated with subsequent 3 years cognitive decline. The protective factors were the presence of dyslipidemia and a higher educational level. Therefore, these factors are considered important and should be taken into consideration when searching for creative solutions to prevent cognitive decline after stroke in community dwelling older and oldest people.

## Background

Recently, Japan has become a super-aged society, among the first in the world [1]. Cognitive impairment is a major health concern among older people as it threatens independent and active life, and eventually survival [2]. As cognitive impairment and dementia have been identified as the strongest independent predictor of medical and long-term care (LTC) utilization and expenditure among older and oldest individuals. Therefore, identifying relevant risk factors of cognitive impairment and dementia have to be addressed. Research performed has shown the relevant contribution of vascular risk factors to the development of dementia and stroke is the second most common cause of dementia [3]. The World Health Organization definition of stroke is “rapidly developing clinical signs of focal (or global) disturbance of cerebral function, with symptoms lasting 24 hours or longer or leading to death, with no apparent cause other than of vascular origin” [4]. In general, the incidence of stroke increases with age, occurring in up to 69% of individuals older than 65 years and 34.4% in those older than 75 years [5].

Due to an increase in the older population and a decline in mortality after stroke, the rate of post-stroke cognitive decline has increased [6, 7]. Moreover, cognitive decline can happen both immediately and long after the incidence of stroke [8]. Therefore, understanding stroke as a risk factor of cognitive decline is necessary so that preventive strategies against cognitive impairment and dementia, especially in older and oldest people, can be identified. The risk factors of cognitive decline related to stroke are numerous, as reported in previous studies, including: age [9–17], sex [9, 11, 12], vascular risk factors such as hypertension [9, 11, 15, 18], diabetes mellitus [12, 14–16, 19, 20], dyslipidemia [17, 21], atrial fibrillation [22–24], and current smoking [11, 18], and social factors including educational level [9, 11–13, 16, 17], frequency of going outside [25], and LTC service used [26]. The relationship between stroke as a risk factor with cognitive decline reported in previous studies may differ due to cognitive function assessment methods, but the MoCA-J is considered a useful cognitive assessment for detecting mild cognitive impairment (MCI), and its results are used to offer recommendations in a primary clinical setting and for geriatric health screening in the community. Moreover, the MoCA-J has been shown to detect cognitive decline over a 2-year period in older people with MCI and early-stage Alzheimer’s disease [2].

The research on cognitive decline by focusing on the importance of stroke as a risk factor in those 80 years old or older including the oldest people may have traditionally been overlooked. Most of the previous studies focused on cognitive impairment or stroke in older people separately and studies in oldest people are rare [6, 18, 26]. Moreover, the effect of stroke as a risk factor on cognitive decline in post-stroke who have no deficit symptoms and continued living in the community is not clear. The big question is how cognitive function will be declined by aging, especially in post-stroke older and oldest people with no deficit symptoms. Some studies have been published to compare post-stroke cognitive decline in younger and older stroke patients [22], but few of them have used community-based samples [27, 28]. There is a lack of studies that compared older and oldest people with a history of stroke to people without stroke in a community setting. The issue is relevant, as stroke is the important risk factor for cognitive decline and progression to dementia may be avoided by implementing prevention actions regarding risk factor. Despite this, population-based data on cognitive decline of stroke risk factor are limited and have not explored the entire age range of the older people especially 80 years old and over including oldest old. Thus, the purpose of this study was to investigate the importance of stroke as a risk factor of cognitive decline during 3 years in community dwelling older and oldest people.

## Methods

### Study sample

This study was a longitudinal analysis that collected data at the baseline and 3-year follow-up of a prospective cohort study called the Septuagenarians, Octogenarians, Nonagenarians Investigation with Centenarians (SONIC) study, a study ongoing since 2010 [29]. The participants who were living independently were recruited from residential registries and sent letters inviting them to participate in the venue survey nearby their residential area at the baseline. The setting was two regions of western and eastern Japan, and each region was composed of both urban and rural areas: Itami City, Hyogo (western urban); Asago City, Hyogo (western rural); Itabashi ward, Tokyo (eastern urban); and Nishitama county, Tokyo (eastern rural).

**Fig. 1.**
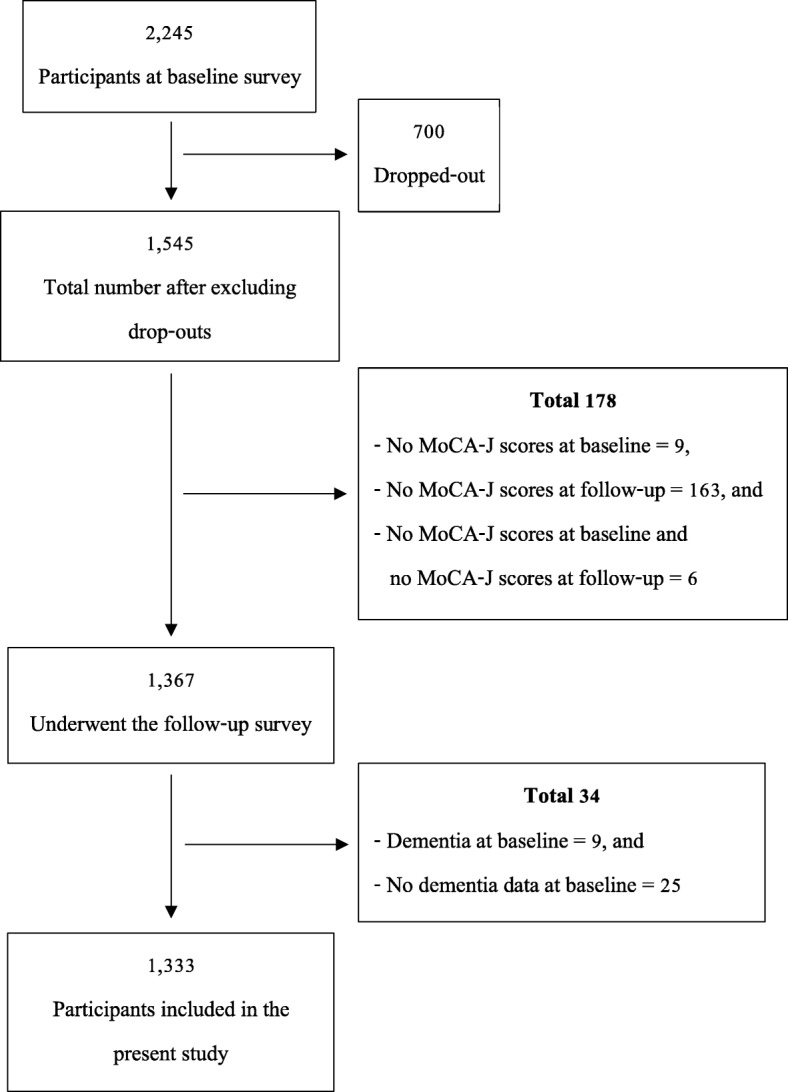
Participants included in the study

The inclusion criteria of this study were as follows: 1) they were free of dementia at the baseline, 2) their completed dementia data were available, and 3) the MoCA-J score was administered both at the baseline and 3-year follow-up. The exclusion criteria were the participants who cannot participate in the consecutive 3-year at the survey venue due to severe disability/death/move to other areas including more severe due to stroke or recent stroke. All data were collected at the baseline (2010–2012) while MOCA-J was performed both at the baseline and the 3-year follow-up (2013–2015). At the baseline, a total of 2245 participants in all age groups (69–71 years old = 1000, 79–81 years old = 973, and 89–91 years old = 272) were included, but only 1333 participants met the inclusion criteria and completed the 3-year follow-up (Fig. 1).

### Basic data

The basic data were collected on the following variables including age and sex while history of stroke was determined based on interviews by the physicians or nurses at the baseline survey and the participants were classified in accordance with their yes/no responses. Participants were asked what type of stroke they had and classified to ischemic stroke, intracerebral hemorrhage, subarachnoid hemorrhage, subdural hematoma and Transient Ischemic Stroke (TIA).

Information on dementia at baseline was determined based on a combination of self-administered with their yes/no responses and information on dementia drugs at baseline in the participants’ medication record book.

### Vascular risk factors

Blood pressure was measured by a physician and trained nurses. A sphygmomanometer was used to measure blood pressure twice with each arm in a sitting position. The average of the first and second measurements of each arm was used in the analysis. Hypertension was diagnosed according to the Japanese Society of Hypertension guideline 2019 [30], which is defined by systolic Blood Pressure (BP) ≥ 140 mmHg and diastolic BP ≥ 90 mmHg or the use of antihypertensive drugs at the first survey.

The blood samples were collected for subsequent analysis. The levels of fasting/casual blood glucose, low-density lipoprotein (LDL) cholesterol, high-density lipoprotein (HDL) cholesterol, and triglycerides were determined using biochemical examinations. Diabetes mellitus was defined by fasting blood glucose ≥126 mg/dL, casual blood glucose ≥200 mg/dL, hemoglobin A1c ≥ 6.5%, or use of antidiabetic drugs according to the Japan Diabetes Society [31]. Dyslipidemia was defined by LDL-cholesterol ≥140 mg/dL, HDL-cholesterol < 40 mg/dL, triglycerides ≥150 mg/dL, or use of dyslipidemia drugs according to the Japan Atherosclerosis Society [32]. Finally, atrial fibrillation was determined with a self-administered questionnaire with yes/no answers.

Current smoking behavior was determined based on a self-administered questionnaire and the participants were classified in accordance with their yes/no responses.

### Social factors

Data were collected on the following variables through self-administered questionnaires at the baseline survey: educational level (< 10 years [junior high school or less], 10–12 years [high school], or > 12 years [university or higher]), frequency of going outdoors (< 1 time/week, 1 or 2 times per week, 3 or 4 times per week, 5 or 6 times per week, and every day). The participants were asked whether they had used LTC services with a mail questionnaire. Finally, residential area was collected based on the residential registries and classified to urban or rural areas.

### Assessment of cognitive functioning

The participants’ cognitive function using the MoCA-J [2] was performed by trained psychologists. The MoCA-J total scores (0–30 points) were used for cognitive function assessment. A higher score indicated a higher cognitive function. Generally, the MoCA-J demonstrates greater reliability and validity in the screening of MCI in community-dwelling older people than conventional cognitive tests.

The MoCA-J scores at the 3-year follow-up subtracted from the scores at the baseline were used to define changes in the MoCA-J scores. Therefore, the participants whose MoCA-J scores decreased by ≥2 points were defined as those with cognitive decline, while the participants whose scores decreased by < 2 points were defined as those with maintained cognition [33, 34].

### Statistical analysis

After the computation of summary statistics, the Pearson’s Chi-square or Fisher’s exact test for categorical variables and the independent t-test for continuous variables were employed to compare baseline characteristics between stroke and non-stroke, maintained cognition and cognitive decline groups (based on changes in MoCA-J scores), and follow-up and dropped-out groups. Cognitive decline (MoCA-J scores deceased by ≥2 points) was considered the outcome variable.

Logistic regression analysis was used to determine the association, expressed as an odds ratio (OR) and 95% confidence interval (CI), between risk factors and cognitive decline. Univariate logistic regression was tested for age, sex, and MoCA-J score at the baseline. In addition, multiple logistic regression was implemented in model 1 with each variable being adjusted by age, sex, and MoCA-J score at the baseline and model 2 was adjusted by all variables (age, sex, MoCA-J score at the baseline, history of stroke, hypertension, diabetes mellitus, dyslipidemia, atrial fibrillation, current smoking, educational level, frequency of going outdoors, LTC service used, and residential areas). These statistical analyses were carried out with SPSS Statistics 24.0 (IBM Japan, Tokyo, Japan). Significance was set at .05.

## Results

**Fig. 2.**
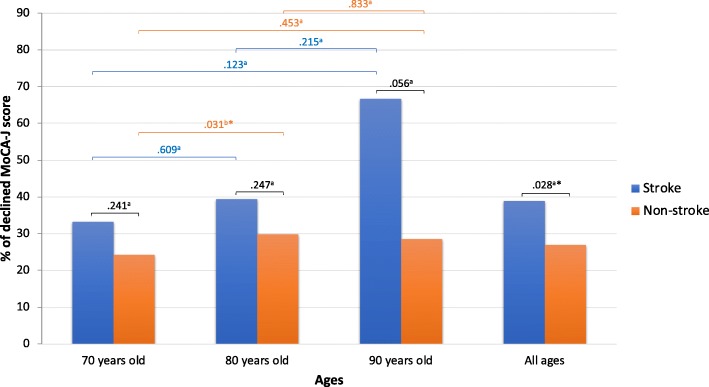
Percentage of declined MoCA-J scores in the stroke and non-stroke groups. a *P*-values from Pearson’s Chi-square test; b *P*-values from Fisher’s exact test; * *P*-values <.05

There were 2245 participants recruited at the baseline survey and 1333 (59.38%) met the inclusion criteria and completed the 3-year follow-up, as shown in Fig. 1. Half of the participants were 70 years old (675 participants or 50.6%). Almost half of the participants were male (657 participants or 49.3%). There were 72 participants (5.4%) who had a history of stroke. Furthermore, when the participants were divided based on a history of stroke, there were significant differences between the participants who had and did not have stroke in terms of hypertension, diabetes mellitus, atrial fibrillation, frequency of going outdoors, use of LTC services and MoCA-J score at the follow-up (Table 1). Details of each age group are shown in Additional file 1: Table S1. Moreover, only a history of stroke, MoCA-J score at the baseline, and MoCA-J at the follow-up in all age groups showed significant differences when a comparison was made between those with unchanged cognition and those with cognitive decline at the baseline (Additional file 2: Table S2). However, a history of stroke, diabetes mellitus, current smoking, educational level, frequency of going outdoors, LTC service used, residential areas, and MoCA-J score at the baseline were significantly different when comparing the follow-up and dropped-out groups at the baseline, which may indicate some selection bias in this study (Additional file 3: Table S3). Also, the percentage of stroke participants with cognitive decline increased with age, equaling 33.3, 39.4, and 66.7% in those who were 70, 80, and 90 years old, respectively. The MoCA-J score decline showed a significant difference between stroke and non-stroke groups in all ages (*P* < .028). Comparing the MoCA-J score decline between stroke and non-stroke groups in each age group (70, 80, and 90 years old), there was no significant difference. In comparison, the MoCA-J score decline between ages (stroke vs. non-stroke) revealed a significant difference only in the non-stroke group between 70 and 80 years old (*P* < .031) (Fig. 2). In addition, in this study the strength was included participants with 80 years old and older. So, sub-analysis to compare those between < 80 years old and ≥ 80 years old was performed in comparison of maintained/declined and stroke/non-stroke groups as shown in Additional file 5: Table S5 and Additional file 6: Table S6, respectively.

**Table 1:** Comparison of a history of stroke as a baseline characteristic (*n* = 1333)

Characteristics	Total n (%)	Stroke *n* = 72 (5.4%)	Non-stroke *n* = 1261 (94.6%)	*P*-value
Age, %				
70 years old	675 (50.6)	45.8	50.9	.395^a^
80 years old	589 (44.2)	45.8	44.1	
90 years old	69 (5.2)	8.3	5.0	
Sex, %				
Male	657 (49.3)	59.7	48.7	.070^b^
Female	676 (50.7)	40.3	51.3	
Hypertension, %				
No	343 (26.1)	12.9	26.9	.009^a^
Yes	969 (73.9)	87.1	73.1	
Diabetes mellitus, %				
No	1054 (84.9)	75.8	85.4	.034^a^
Yes	188 (15.1)	24.2	14.6	
Dyslipidemia, %				
No	504 (38.8)	31.9	39.2	.254^b^
Yes	796 (61.2)	68.1	60.8	
Atrial fibrillation, %				
No	1304 (97.8)	93.1	98.1	.004^a^
Yes	29 (2.2)	6.9	1.9	
Current smoking, %				
No	1165 (89.0)	94.4	88.7	.137^a^
Yes	144 (11.0)	5.6	11.3	
Educational level, %				
< 10 years	354 (26.6)	23.9	26.8	.771^a^
10–12 years	565 (42.5)	46.5	42.3	
> 12 years	410 (30.9)	29.6	30.9	
Frequency of going outdoors, %				
< 1 time/week	79 (5.9)	5.6	12.5	.043^a^
1–2 times/week	169 (12.7)	12.4	18.1	
3 or 4 times/week	275 (20.7)	20.7	20.8	
5 or 6 times/week	271 (20.4)	20.8	12.5	
Every day	535 (40.3)	40.5	36.1	
LTC service used, %				
No	1220 (95.9)	91.3	96.2	.047^a^
Yes	52 (4.1)	8.7	3.8	
Residential areas, %				
Urban	788 (59.1)	56.9	59.2	.713^b^
Rural	545 (40.9)	43.1	40.8	
MoCA-J score at the baseline, Mean ± SD	23.04 ± 3.50	22.57 ± 3.91	23.07 ± 3.48	.242
MoCA-J score at the follow-up, Mean ± SD	23.03 ± 3.90	22.13 ± 4.94	23.09 ± 3.83	.042

^a^ *P*-values from Pearson’s Chi-square test. ^b^ *P*-values from Fisher’s exact test for categorical variables and independent t-test for continuous variable

Multiple logistic regression by adjusting for age, sex, and MoCA-J score at the baseline showed that a history of stroke (OR = 1.88) was an independent risk factor of cognitive decline, while a higher educational level (10–12 years, OR = 0.63, > 12 years, OR = 0.55, with < 10 years as a reference) was a protective factor significantly correlated with cognitive decline at the 3-year follow-up. Moreover, after adjusting for all variables, the results revealed that a history of stroke (OR = 1.83), advanced age (80 years old, OR = 2.12; 90 years old, OR = 4.09, with 70 years old as a reference), and MoCA-J score at the baseline (OR = 1.26) were independent risk factors, whereas the presence of dyslipidemia (OR = 0.71) and a higher educational level (10–12 years, OR = 0.61, > 12 years, OR = 0.52, with < 10 years as a reference) were protective factors significantly correlated with cognitive decline at the 3-year follow-up (Table 2).

**Table 2:** Logistic regression model

Characteristics	Model 1 Adjusted age, sex, MoCA-J scores at baseline		Model 2^c^ (All adjusted)	
	OR (95% CI)	*P*-value	OR (95% CI)	*P*-value
Age (reference; 70 years old)^a^				
80 years old	1.33 (1.04–1.70)	.025	2.12 (1.54–2.91)	<.001
90 years old	1.42 (0.83–2.43)	.196	4.09 (2.08–8.04)	<.001
Sex^a^ (reference; male)	1.10 (0.87–1.40)	.435	1.14 (0.85–1.53)	.387
MoCA-J score at the baseline^a^	1.18 (1.13–1.23)	<.001	1.26 (1.20–1.33)	<.001
History of stroke^b^	1.88 (1.11–3.16)	.018	1.83 (1.01–3.31)	.046
Hypertension^b^	0.98 (0.73–1.31)	.871	1.04 (0.75–1.44)	.815
Diabetes mellitus^b^	1.37 (0.97–1.96)	.077	1.37 (0.94–2.01)	.102
Dyslipidemia^b^	0.78 (0.60–1.01)	.061	0.71 (0.53–0.94)	.019
Atrial fibrillation^b^	0.94 (0.40–2.21)	.889	1.04 (0.43–2.56)	.925
Current smoking^b^	1.30 (0.86–1.97)	.213	1.44 (0.90–2.32)	.131
Educational level^b^ (reference < 10 years)				
10–12 years	0.63 (0.46–0.87)	.005	0.61 (0.43–0.86)	.005
> 12 years	0.55 (0.39–0.78)	.001	0.52 (0.35–0.77)	.001
Frequency of going outdoors^b^ (reference; < 1 time/week)				
1–2 times/week	0.95 (0.50–1.79)	.874	1.10 (0.54–2.23)	.802
3 or 4 times/week	0.92 (0.51–1.68)	.792	1.02 (0.52–2.00)	.966
5 or 6 times/week	0.88 (0.48–1.60)	.667	1.21 (0.61–2.39)	.592
Every day	0.84 (0.47–1.49)	.550	0.98 (0.51–1.89)	.946
LTC service used^b^	1.19 (0.60–2.36)	.612	1.01 (0.48–2.16)	.975
Residential areas (reference; urban)	1.18 (0.91–1.53)	.207	1.09 (0.80–1.47)	.590

^a^ univariate logistic regression analysis

^b^ adjusted for age, sex, and MoCA-J score at the baseline

^c^ all adjusted variables

## Discussion

The present longitudinal study demonstrated that the prevalence of stroke in community dwelling older and oldest people was 5.4%. On the other hand, the prevalence of cognitive decline after the 3-year follow-up in this survey increased in advanced ages (33.3, 39.4, and 66.7% in those who were 70, 80, and 90 years old, respectively). Previous studies reported that the prevalence of post-stroke cognitive impairment ranges widely from 20 to 80% [7, 35–37]. However, the prevalence of stoke in this study was low because post-stroke participants who had no deficit symptoms were able to participate in the investigation but those who still had deficit symptoms were unable to take part in this study. Even though there were a small number of stroke participants, the findings shed more light on cognitive decline in older and oldest people. Moreover, our results demonstrate that participants who developed cognitive decline could be classified into two major groups based on risk factors including a history of stroke and advanced age, while greater MoCA-J score at the baseline was positively associated with subsequent 3-year cognitive decline. The protective factors including the presence of dyslipidemia and a higher educational level.

The main finding of the present study was the association between the history of stroke and cognitive decline after the 3-year follow-up. One plausible explanation is that stroke and cognitive decline are common among older persons [38]. As this study included the oldest group of the population, the prevalence of cognitive decline after stroke may have increased and may have had continuous effects that were evidenced during the follow-up. Therefore, an advanced age was considered a risk factor of not only stroke but also cognitive decline [39]. Therefore, a combination of stroke and aging will result in a strong risk factor for stroke patients to develop cognitive decline. The risk factor of post-stroke cognitive decline in this study increased gradually by approximately one-fold with an age increase of 1 year, which was consistent with the study of Renjen PN, et al. [40], who found that the prevalence of cognitive decline was higher with increasing age (100% in an 80–89-year-old age group). In terms of the biological mechanism, β-amyloid deposition, one of the pathological hallmarks of Alzheimer’s disease, may play a significant role in cognitive dysfunction associated with aging, and in cognitively normal older people it may be associated with gray matter atrophy and memory impairment [41]. The role of stroke pathology, besides neurodegenerative abnormalities, in the aging process, and a synergistic role for these two components, has been documented [42, 43].

Moreover, in the present study, the MoCA-J score at the baseline was associated with cognitive decline. We found that an increase in the MoCA-J score at the baseline by one point increased cognitive decline by approximately one-fold. Besides this, older people who had higher MoCA-J scores at the baseline likely developed cognitive decline. In one conventional Mini-mental Status Examination (MMSE) study, patients with a high cognitive function showed a greater reduction in MMSE than patients with a low cognitive function, which was at odds with most population studies [44]. The results of the present study suggest that stroke patients with low MoCA-J scores at the baseline were already on a trajectory of cognitive decline related to pre-existing neurodegenerative lesions or other undetermined factors triggered by the initial stroke event [45].

On the other hand, dyslipidemia was found to be a protective factor in the present study, which means that the participants who were diagnosed with dyslipidemia had a decreased risk of cognitive decline after stroke. This finding is similar to the Framingham Heart Study found high cholesterol levels were associated with improved cognitive function [46]. In contrast, a longitudinal study of 1159 elderly Chinese individuals found associations between the elevated levels of total cholesterol and low-density lipoprotein (LDL) and the accelerated cognitive decline [47]. The relationship between plasma lipids and cognition is very complex, controversial, and still unclear recently. Consequently, treatment of dyslipidemia would be expected to reduce the risk of dementia. Strongest support for a beneficial effect of statin treatment comes from a recently published large numbered observational study with 2 years of follow-up, which showed that simvastatin users had a lower risk of dementia than non-users [48]. Opposing this result was the ‘Heart Protection Study’, which was performed in subjects in an age-range from 40 to 80 years, showed that allocation to statin treatment did not decrease the risk of developing dementia during follow-up [49]. So, the effect of statin on cognitive function still controversial and unclear as dyslipidemia condition and need more elucidate regarding these issues. In addition, in the present study, the result found that statins did not reduce risk of cognitive decline as shown in Additional file 7: Table S7.

The higher educational level was also a protective factor in this study. This result may explain by the higher education is associated with a lower risk of cognitive decline and dementia, which is thought to be explained by two major probable explanations. Firstly, cognitive reserve is thought to be built by cognitively enduring activities such as education and occupation complexity [50]. The cognitive reserve hypothesis postulates that people with a higher reserve can tolerate more neurodegenerative pathology and maintain brain function for longer than people with low reserve, before the damage manifests clinically as dementia [51]. Secondly, an alternative explanation is that people with higher education have a more favorable environment including a healthier lifestyle, better compliance to treatment and better access to healthcare leading to less cognitive decline and dementia [50]. Moreover, we focused stroke as the important risk factor of cognitive decline, as we known that people with stroke have an increased risk of cognitive decline and dementia compared to those without stroke. The study of Mirza et al. [50] indicated that stroke increased the risk of subsequent cognitive decline and dementia in persons with low and intermediate education but not in those with high education.

A strength of the SONIC study was a longitudinal study investigated in the community dwelling participants including large group of 80 years old and oldest people. This longitudinal study with narrow age ranges may investigate the differences in the influence of aging on the importance of stroke as a risk factor of cognitive decline in later life. Moreover, in our SONIC study we assess cognitive function by using MoCA-J which is exhibiting a comparatively normal distribution and it was more sensitive to detect MCI than conventional MMSE. Finally, this study was involved a multidisciplinary team (medicine, dentist, nurse, nutritionist, and psychologist) to collect data with reliability including wide range of important confounding factors in a high-quality level.

In the present study, several limitations have to be mentioned. Firstly, the participants could not be considered representative of the general older Japanese population. Therefore, there was a possibility of selection bias. Furthermore, the numbers of participants with a history of stroke and cognitive decline events were small. These might, to some extent, limit the external validity of the findings. Secondly, the population included only non-institutionalized community dwelling Japanese people in limited areas. Consequently, our results cannot be generalized to younger, older, or less healthy people with a history of stroke. Thirdly, the data regarding stroke history and onset had low objective reliability because they were obtained from personal interviews. Comprehensive medical and imaging examinations to diagnose stroke may have yielded more precise and accurate results. Moreover, information regarding location and severity of stroke including length of hospital stay and rehabilitation period were lacking which may also in cognitive decline concerns. Fourthly, this study was longitudinal in nature, so we were unable to implement a direct comparison between the participants in the study at endpoints because of subject loss during the follow-up, severe disability, or death. Moreover, the stroke ratio between follow-up and dropped-out groups was equal (1:1) and the ratio between with and without a history of stroke in the follow-up group was higher than in the dropped-out group (1:17.51 vs. 1:11.67, respectively) (Additional file 4: Table S4) and the MoCA-J score at the baseline in the follow-up group was greater than in the dropped-out group in each age group and all age groups combined, with significant differences. Thus, participants in the follow-up examination of the present study represent a relatively healthy group. This might have led to an underestimation of the true relationship between stroke and cognitive decline. Fifthly, there was no precise cut-off point for a significant decline in MoCA-J scores. Therefore, the present study specified the cut-off value for cognitive decline at the 3-year follow-up by reduction of 2 points or greater of MoCA-J scores subtracted from the scores at the baseline [33, 34]. Sixthly, we used self-reported questionnaires to obtain data regarding the frequency of going outdoors as the indicator of physical activity. This may be a crude measure compared with energy expenditure. In fact, objective measures such as digital devices that automatically measure the activity level, walking speed, and periods of activity should have been used instead. Finally, since we could not perform electrocardiography to diagnose atrial fibrillation and had to rely on personal interviews, the data obtained may have not been as accurate.

## Conclusions

Several studies found various risk factors associated with cognitive decline, but this study is the first investigation involving community dwelling with larger group of 80 years old and older in Japan. Moreover, the present study focused on the important of stroke as a risk factor of cognitive decline. The result in the current study indicates that a history of stroke, and advanced age were risk factors while the presence of dyslipidemia and higher educational level were protective factors during the 3-year follow-up. Moreover, while greater MoCA-J score at the baseline was positively associated with subsequent 3-year cognitive decline. The recommendation to prevent cognitive decline by focusing on the stroke risk factors is very important in older and oldest people. So, primary prevention by eliminating or prevent risk factors of stroke for further develop of cognitive decline such in this study including hypertension, diabetes, dyslipidemia, and atrial fibrillation especially life-style modification is the huge issue for reduction of incidence of stroke related risk factors. Finally, secondary prevention for recurrence stroke by avoiding stroke risk factors and continue treatment advice by the physician is a crucial implementation.

## Supplementary information

**Additional file 1: Table S1.** Comparison of a history of stroke as a baseline characteristic (*n* = 1333).

**Additional file 2: Table S2.** Comparison of baseline characteristics between those with maintained and declined MoCA-J scores (*n* = 1333).

**Additional file 3: Table S3.** Comparison of baseline characteristics between follow-up and dropped-out groups (*n* = 2033).

**Additional file 4: Table S4.** Comparison of stroke and non-stroke ratio between follow-up and dropped-out groups.

**Additional file 5: Table S5.** Comparison of baseline characteristics between those with maintained and declined MoCA-J scores between age < 80 years old and age ≥ 80 years old (*n* = 1333).

**Additional file 6: Table S6.** Comparison of baseline characteristics between stroke and non-stroke groups in age < 80 years old and age ≥ 80 years old (n = 1333).

**Additional file 7: Table S7.** Statin used in both stroke/non-stroke and maintained/declined groups (*n* = 1333).

## Data Availability

The datasets used and/or analyzed during the current study are available from the corresponding author on reasonable request.

## Abbreviations

BP: Blood pressure
CI: Confidence interval
HDL: High-density lipoprotein
LDL: Low-density lipoprotein
LTC: Long-term care
MCI: Mild cognitive impairment
mg/dL: Milligrams per deciliter
MMSE: Mini-mental Status Examination
MoCA-J: The Japanese version of the Montreal Cognitive Assessment
OR: Odds ratio
SONIC: The Septuagenarains, Octogenarians, Nonagenarians Investigation with Centenarians study
TIA: Transient ischemic stroke
