# Supplementary material for: The importance of stroke as a risk factor of cognitive decline in community dwelling older and oldest peoples: the SONIC study
Source: BMC Geriatr. 2020 Jan 22;20:24. doi: 10.1186/s12877-020-1423-5 (PMC6977260; doi:10.1186/s12877-020-1423-5)
Supplement: Supplementary file 1 — Additional file 1: Table S1. Comparison of a history of stroke as a baseline characteristic (n = 1333). [file 12877_2020_1423_MOESM1_ESM.doc]

**Additional file 1: Table S1.** Comparison of a history of stroke as a baseline characteristic (n=1,333)

| **Characteristics** | **Total**  **n (%)** | **70 years old** | | | **80 years old** | | | **90 years old** | | |
| --- | --- | --- | --- | --- | --- | --- | --- | --- | --- | --- |
|  |  | **Stroke**  **n=33**  **(4.9%)** | **Non-stroke**  **n=642**  **(95.1%)** | ***P*-value^a^** | **Stroke**  **n=33**  **(5.6%)** | **Non-stroke**  **n=556**  **(94.4%)** | ***P*-value^a^** | **Stroke**  **n=6**  **(8.7%)** | **Non-stroke**  **n=63**  **(91.3%)** | ***P*-value^a^** |
| **Sex,** % |  |  |  |  |  |  |  |  |  |  |
| Male | 657 (49.3) | 54.5 | 47.8 | .451 | 66.7 | 49.6 | .057 | 50.00 | 49.2 | .970 |
| Female | 676 (50.7) | 45.5 | 52.2 |  | 33.3 | 50.4 |  | 50.00 | 50.8 |  |
| **Hypertension,** % |  |  |  |  |  |  |  |  |  |  |
| No | 343 (26.1) | 19.4 | 35.6 | .064 | 9.1 | 18.2 | .183 | 0.0 | 16.4 | .282 |
| Yes | 969 (73.9) | 80.6 | 64.4 |  | 90.9 | 81.8 |  | 100.0 | 83.6 |  |
| **Diabetes mellitus,** % |  |  |  |  |  |  |  |  |  |  |
| No | 1054 (84.9) | 73.3 | 84.7 | .095 | 76.7 | 86.8 | .116 | 83.3 | 79.0 | .804 |
| Yes | 188 (15.1) | 26.7 | 15.3 |  | 23.3 | 13.2 |  | 16.7 | 21.0 |  |
| **Dyslipidemia,** % |  |  |  |  |  |  |  |  |  |  |
| No | 504 (38.8) | 26.7 | 38.3 | .200 | 36.4 | 40.1 | .673 | 33.3 | 39.7 | .761 |
| Yes | 796 (61.2) | 73.3 | 61.7 |  | 63.6 | 59.9 |  | 66.7 | 60.3 |  |
| **Atrial fibrillation,** % |  |  |  |  |  |  |  |  |  |  |
| No | 1304 (97.8) | 97.0 | 98.4 | .515 | 90.9 | 97.7 | .020 | 83.3 | 98.4 | .035 |
| Yes | 29 (2.2) | 3.0 | 1.6 |  | 9.1 | 2.3 |  | 16.7 | 1.6 |  |
| **Current smoking,** % |  |  |  |  |  |  |  |  |  |  |
| No | 1165 (89.0) | 93.9 | 82.9 | .095 | 93.9 | 94.7 | .855 | 100.0 | 96.5 | .670 |
| Yes | 144 (11.0) | 6.1 | 17.1 |  | 6.1 | 5.3 |  | 0.0 | 3.5 |  |
| **Educational level,** % |  |  |  |  |  |  |  |  |  |  |
| < 10 years | 354 (26.6) | 28.1 | 30.6 | .665 | 15.2 | 28.8 | .149 | 33.3 | 34.9 | .969 |
| 10-12 years | 565 (42.5) | 40.6 | 45.2 |  | 54.5 | 39.6 |  | 33.3 | 36.5 |  |
| > 12 years | 410 (30.9) | 31.3 | 24.2 |  | 30.3 | 31.5 |  | 33.3 | 28.6 |  |
| **Frequency of going outdoors,** % |  |  |  |  |  |  |  |  |  |  |
| < 1 time/week | 79 (5.9) | 12.1 | 3.9 | .066 | 15.2 | 6.7 | .351 | 0.0 | 12.9 | .594 |
| 1 or 2 times/week | 169 (12.7) | 15.2 | 8.9 |  | 21.2 | 16.2 |  | 16.7 | 14.5 |  |
| 3 or 4 times/week | 275 (20.7) | 18.2 | 18.0 |  | 18.2 | 23.6 |  | 50.0 | 22.6 |  |
| 5 or 6 times/week | 271 (20.4) | 9.1 | 22.5 |  | 15.2 | 18.9 |  | 16.7 | 21.0 |  |
| Every day | 535 (40.3) | 45.5 | 46.6 |  | 30.3 | 34.7 |  | 16.7 | 29.0 |  |
| **LTC service used,** % |  |  |  |  |  |  |  |  |  |  |
| No | 1220 (95.9) | 100.0 | 98.5 | .496 | 90.9 | 95.3 | .265 | 50.0 | 82.5 | .058 |
| Yes | 52 (4.1) | 0.0 | 1.5 |  | 9.1 | 4.7 |  | 50.0 | 17.5 |  |
| **Residential areas,** % |  |  |  |  |  |  |  |  |  |  |
| Urban | 788 (59.1) | 51.5 | 55.1 | .683 | 60.6 | 63.5 | .738 | 66.7 | 63.5 | .877 |
| Rural | 545 (40.9) | 48.5 | 44.9 |  | 39.4 | 36.5 |  | 33.3 | 36.5 |  |
| **MoCA-J score at the baseline,** Mean±SD | 23.04±3.50 | 23.64±  3.57 | 23.99±  3.07 | .526 | 21.70±  4.10 | 22.40±  3.49 | .269 | 21.50±  3.78 | 19.59±  3.75 | .237 |
| **MoCA-J score at the follow-up,** Mean±SD | 23.03±3.90 | 23.06±  5.27 | 24.07±  3.37 | .103 | 21.45±  4.62 | 22.38±  3.82 | .183 | 20.67±  4.50 | 19.25±  4.55 | .470 |

Abbreviation: LTC, long-term care; MoCA-J, the Japanese version of the Montreal Cognitive Assessment; SD, Standard Deviation.

^a^ *P*-values from Pearson’s Chi-square test for categorical variables and independent t-test for continuous variable
