# Supplementary material for: The importance of stroke as a risk factor of cognitive decline in community dwelling older and oldest peoples: the SONIC study
Source: BMC Geriatr. 2020 Jan 22;20:24. doi: 10.1186/s12877-020-1423-5 (PMC6977260; doi:10.1186/s12877-020-1423-5)
Supplement: Supplementary file 2 — Additional file 2: Table S2. Comparison of baseline characteristics between those with maintained and declined MoCA-J scores (n = 1333). [file 12877_2020_1423_MOESM2_ESM.doc]

**Additional file 2: Table S2.** Comparison of baseline characteristics between those with maintained and declined MoCA-J scores (n=1,333)

| **Characteristics** | **Total**  **n (%)** | **70 years old** | | | **80 years old** | | | **90 years old** | | | **All ages** | | |
| --- | --- | --- | --- | --- | --- | --- | --- | --- | --- | --- | --- | --- | --- |
|  |  | **Maintained**  **n=508**  **(75.3%)** | **Declined**  **n=167**  **(24.7%)** | ***P*-value** | **Maintained**  **n=410**  **(69.6%)** | **Declined**  **n=179**  **(30.4)** | ***P*-value** | **Maintained**  **n=47**  **(68.1%)** | **Declined**  **n=22**  **(31.9%)** | ***P*-value** | **Maintained**  **n=965**  **(72.4%)** | **Declined**  **n=368**  **(27.6%)** | ***P*-value** |
| **History of stroke,** % |  |  |  |  |  |  |  |  |  |  |  |  |  |
| No | 1261 (94.6) | 95.7 | 93.4 | .241^a^ | 95.1 | 92.7 | .247^a^ | 95.7 | 81.8 | .056^a^ | 95.4 | 92.4 | .028^a^ |
| Yes | 72 (5.4) | 4.3 | 6.6 |  | 4.9 | 7.3 |  | 4.3 | 18.2 |  | 4.6 | 7.6 |  |
| **Sex,** % |  |  |  |  |  |  |  |  |  |  |  |  |  |
| Male | 657 (49.3) | 48.6 | 46.7 | .721^b^ | 51.2 | 49.2 | .655^b^ | 53.2 | 40.9 | .342^a^ | 49.9 | 47.6 | .462^b^ |
| Female | 676 (50.7) | 51.4 | 53.3 |  | 48.8 | 50.8 |  | 46.8 | 59.1 |  | 50.1 | 52.4 |  |
| **Hypertension,** % |  |  |  |  |  |  |  |  |  |  |  |  |  |
| No | 343 (26.1) | 34.1 | 37.0 | .507^b^ | 17.4 | 18.4 | .814^b^ | 17.8 | 9.1 | .349^a^ | 26.1 | 26.2 | 1.000^b^ |
| Yes | 969 (73.9) | 65.9 | 63.0 |  | 82.6 | 81.6 |  | 82.2 | 90.9 |  | 73.9 | 73.8 |  |
| **Diabetes mellitus,** % |  |  |  |  |  |  |  |  |  |  |  |  |  |
| No | 1054 (84.9) | 86.2 | 77.7 | .019^b^ | 85.6 | 87.7 | .594^b^ | 82.6 | 72.7 | .346^a^ | 85.8 | 82.4 | .156^b^ |
| Yes | 188 (15.1) | 13.8 | 22.3 |  | 14.4 | 12.3 |  | 17.4 | 27.3 |  | 14.2 | 17.6 |  |
| **Dyslipidemia,** % |  |  |  |  |  |  |  |  |  |  |  |  |  |
| No | 504 (38.8) | 36.4 | 41.9 | .218^b^ | 39.4 | 41.0 | .715^b^ | 36.2 | 45.5 | .461^a^ | 37.7 | 41.7 | .201 |
| Yes | 796 (61.2) | 63.6 | 58.1 |  | 60.6 | 59.0 |  | 63.8 | 54.5 |  | 62.3 | 58.3 |  |
| **Atrial fibrillation,** % |  |  |  |  |  |  |  |  |  |  |  |  |  |
| No | 1304 (97.8) | 98.4 | 98.2 | .844^a^ | 97.1 | 97.8 | .635^a^ | 97.9 | 95.5 | .577^a^ | 97.8 | 97.8 | 1.000^b^ |
| Yes | 29 (2.2) | 1.6 | 1.8 |  | 2.9 | 2.2 |  | 2.1 | 4.5 |  | 2.2 | 2.2 |  |
| **Current smoking,** % |  |  |  |  |  |  |  |  |  |  |  |  |  |
| No | 1165 (89.0) | 82.9 | 84.8 | .630^b^ | 96.5 | 90.3 | .002^a^ | 95.3 | 100.0 | .339^a^ | 89.3 | 88.3 | .622^b^ |
| Yes | 144 (11.0) | 17.1 | 15.2 |  | 3.5 | 9.7 |  | 4.7 | 0.0 |  | 10.7 | 11.7 |  |
|  |  |  |  |  |  |  |  |  |  |  |  |  |  |
| **Educational level,** % |  |  |  |  |  |  |  |  |  |  |  |  |  |
| < 10 years | 410 (30.9) | 24.8 | 23.8 | .916^a^ | 25.9 | 33.0 | .061^a^ | 36.2 | 31.8 | .918^a^ | 25.8 | 28.8 | .268^a^ |
| 10-12 years | 565 (42.5) | 44.5 | 46.3 |  | 39.9 | 41.9 |  | 36.2 | 36.4 |  | 42.1 | 43.6 |  |
| > 12 years | 354 (26.6) | 30.7 | 29.9 |  | 34.2 | 25.1 |  | 27.7 | 31.8 |  | 32.1 | 27.7 |  |
| **Frequency of going outdoors**, % |  |  |  |  |  |  |  |  |  |  |  |  |  |
| < 1 time/week | 79 (5.9) | 4.7 | 3.0 | .619^a^ | 7.1 | 7.3 | .679^a^ | 13.0 | 9.1 | .180^a^ | 6.1 | 5.5 | .751^a^ |
| 1 or 2 times/week | 169 (12.7) | 8.7 | 10.9 |  | 17.3 | 14.5 |  | 10.9 | 22.7 |  | 12.5 | 13.4 |  |
| 3 or 4 times/week | 275 (20.7) | 17.6 | 19.4 |  | 22.9 | 24.0 |  | 28.3 | 18.2 |  | 20.4 | 21.6 |  |
| 5 or 6 times/week | 271 (20.4) | 21.3 | 23.6 |  | 17.3 | 21.8 |  | 26.1 | 9.1 |  | 19.8 | 21.9 |  |
| Every day | 535 (40.3) | 47.7 | 43.0 |  | 35.4 | 32.4 |  | 21.7 | 40.9 |  | 41.2 | 37.7 |  |
| **LTC service used,** % |  |  |  |  |  |  |  |  |  |  |  |  |  |
| No | 1220 (95.9) | 98.7 | 98.1 | .565^a^ | 94.3 | 96.6 | .241^a^ | 83.0 | 72.7 | .324^a^ | 96.0 | 95.8 | .878^a^ |
| Yes | 52 (4.1) | 1.3 | 1.9 |  | 5.7 | 3.4 |  | 17.0 | 27.3 |  | 4.0 | 4.2 |  |
| **Residential areas,** % |  |  |  |  |  |  |  |  |  |  |  |  |  |
| Urban | 788 (59.1) | 53.9 | 58.1 | .371^b^ | 64.9 | 59.8 | .265^b^ | 61.7 | 68.2 | .602^a^ | 59.0 | 59.5 | .901^b^ |
| Rural | 545 (40.9) | 46.1 | 41.9 |  | 35.1 | 40.2 |  | 38.3 | 31.8 |  | 41.0 | 40.5 |  |
| **MoCA-J score at the baseline,** Mean±SD | 23.04±3.50 | 23.49±  3.06 | 25.43±  2.75 | <.001 | 21.76±  3.38 | 23.72±  3.49 | <.001 | 19.17±  3.90 | 21.00±  3.19 | .044 | 22.55±  3.43 | 24.33±  3.36 | <.001 |
| **MoCA-J score at the follow-up,** Mean±SD | 23.03±3.90 | 24.84±  2.79 | 21.55±  4.17 | <.001 | 23.34±  3.40 | 20.01±  3.88 | <.001 | 20.60±  4.29 | 16.77±  3.99 | .001 | 23.99±  3.32 | 20.51±  4.19 | <.001 |
|  |  |  |  |  |  |  |  |  |  |  |  |  |  |

Abbreviation: LTC, long-term care; MoCA-J, the Japanese version of the Montreal Cognitive Assessment; IQR, interquartile range.

^a^ *P*-values from Person’s Chi-square test. ^b^ *P*-values from Fisher’s exact test for categorical variables and independent t-test for continuous variable.
