# Supplementary material for: The importance of stroke as a risk factor of cognitive decline in community dwelling older and oldest peoples: the SONIC study
Source: BMC Geriatr. 2020 Jan 22;20:24. doi: 10.1186/s12877-020-1423-5 (PMC6977260; doi:10.1186/s12877-020-1423-5)
Supplement: Supplementary file 3 — Additional file 3: Table S3. Comparison of baseline characteristics between follow-up and dropped-out groups (n = 2033). [file 12877_2020_1423_MOESM3_ESM.doc]

**Additional file 3: Table S3.** Comparison of baseline characteristics between follow-up and dropped-out groups (n=2,033)

| **Characteristics** | **Total**  **n (%)** | **70 years old** | | | **80 years old** | | | **90 years old** | | | **All ages** | | |
| --- | --- | --- | --- | --- | --- | --- | --- | --- | --- | --- | --- | --- | --- |
|  |  | **Follow-up**  **n=675**  **(67.9 %)** | **Dropped out**  **n=319**  **(32.1 %)** | ***P*-value** | **Follow-up**  **n=589**  **(72.0 %)** | **Dropped out**  **n=229**  **(28.0 %)** | ***P*-value** | **Follow-up**  **n=69**  **(31.2 %)** | **Dropped out**  **n=152**  **(68.8 %)** | ***P*-value** | **Follow-up**  **n=1333**  **(65.6 %)** | **Dropped out**  **n=700**  **(34.4 %)** | ***P*-value** |
| **History of stroke,** % |  |  |  |  |  |  |  |  |  |  |  |  |  |
| No | 1905 (93.7) | 95.1 | 95.9 | .569^a^ | 94.4 | 90.8 | .065^a^ | 91.3 | 85.5 | .231^a^ | 94.6 | 92.0 | .027^b^ |
| Yes | 128 (6.3) | 4.9 | 4.1 |  | 5.6 | 9.2 |  | 8.7 | 14.5 |  | 5.4 | 8.0 |  |
| **Sex,** % |  |  |  |  |  |  |  |  |  |  |  |  |  |
| Male | 975 (48.0) | 48.1 | 47.0 | .786^b^ | 50.6 | 40.6 | .013^b^ | 49.3 | 49.3 | 1.000^b^ | 49.3 | 45.4 | .102^b^ |
| Female | 1058 (52.0) | 51.9 | 53.0 |  | 49.4 | 59.4 |  | 50.7 | 50.7 |  | 50.7 | 54.6 |  |
| **Hypertension,** % |  |  |  |  |  |  |  |  |  |  |  |  |  |
| No | 507 (24.9) | 34.9 | 30.3 | .168^b^ | 17.7 | 18.1 | .919^b^ | 14.9 | 19.3 | .444^a^ | 26.1 | 24.0 | .304^b^ |
| Yes | 1489 (73.2) | 65.1 | 69.7 |  | 82.3 | 81.9 |  | 85.1 | 80.7 |  | 73.9 | 76.0 |  |
| **Diabetes mellitus,** % |  |  |  |  |  |  |  |  |  |  |  |  |  |
| No | 1555 (76.5) | 84.2 | 74.6 | .001^b^ | 86.3 | 79.4 | .021^b^ | 79.4 | 74.0 | .388^a^ | 84.9 | 76.0 | <.001^b^ |
| Yes | 346 (17.0) | 15.8 | 25.4 |  | 13.7 | 20.6 |  | 20.6 | 26.0 |  | 15.1 | 24.0 |  |
| **Dyslipidemia,** % |  |  |  |  |  |  |  |  |  |  |  |  |  |
| No | 771 (37.9) | 37.3 | 34.1 | .308^b^ | 39.9 | 38.3 | .749^b^ | 39.1 | 52.3 | .081^b^ | 38.8 | 39.6 | .734^b^ |
| Yes | 1203 (59.2) | 62.3 | 65.9 |  | 60.1 | 61.7 |  | 60.9 | 47.7 |  | 61.2 | 60.4 |  |
| **Atrial fibrillation,** % |  |  |  |  |  |  |  |  |  |  |  |  |  |
| No | 1986 (97.7) | 98.4 | 98.1 | .775^a^ | 97.3 | 97.8 | .665^a^ | 97.1 | 95.4 | .552^a^ | 97.8 | 97.4 | .573^a^ |
| Yes | 47 (2.3) | 1.6 | 1.9 |  | 2.7 | 2.2 |  | 2.9 | 4.6 |  | 2.2 | 2.6 |  |
| **Current smoking,** % |  |  |  |  |  |  |  |  |  |  |  |  |  |
| No | 1804 (88.7) | 83.4 | 91.8 | <.001^b^ | 94.6 | 91.9 | .152^a^ | 96.8 | 94.0 | .413^a^ | 89.0 | 92.3 | .018^b^ |
| Yes | 197 (9.7) | 16.6 | 8.2 |  | 5.4 | 8.1 |  | 3.2 | 6.0 |  | 11.0 | 7.7 |  |
| **Educational level,** % |  |  |  |  |  |  |  |  |  |  |  |  |  |
| < 10 years | 634 (31.2) | 24.6 | 39.9 | <.001^a^ | 28.1 | 37.6 | .012^a^ | 34.8 | 46.0 | .157^a^ | 26.6 | 40.5 | <.001^a^ |
| 10-12 years | 832 (40.9) | 44.9 | 39.6 |  | 40.5 | 39.4 |  | 36.2 | 35.3 |  | 42.5 | 38.6 |  |
| > 12 years | 555 (27.3) | 30.5 | 20.6 |  | 31.5 | 23.0 |  | 29.0 | 18.7 |  | 30.9 | 21.0 |  |
| **Frequency of going outdoors,** % |  |  |  |  |  |  |  |  |  |  |  |  |  |
| < 1 time/week | 162 (8.0) | 4.3 | 7.6 | .008^a^ | 7.1 | 9.6 | .078^a^ | 11.8 | 24.7 | .119^a^ | 5.9 | 11.9 | <.001^a^ |
| 1 or 2 times/week | 293 (14.4) | 9.2 | 15.1 |  | 16.5 | 22.3 |  | 14.7 | 16.7 |  | 12.7 | 17.8 |  |
| 3 or 4 times/week | 425 (20.9) | 18.0 | 17.4 |  | 23.3 | 24.5 |  | 25.0 | 26.0 |  | 20.7 | 21.6 |  |
| 5 or 6 times/week | 386 (19.0) | 21.9 | 17.7 |  | 18.7 | 17.9 |  | 20.6 | 12.0 |  | 20.4 | 16.5 |  |
| Every day | 759 (37.3) | 46.6 | 42.3 |  | 34.5 | 25.8 |  | 27.9 | 20.7 |  | 40.3 | 32.2 |  |
| **LTC service used,** % |  |  |  |  |  |  |  |  |  |  |  |  |  |
| No | 1488 (73.2) | 98.6 | 99.1 | .532^a^ | 95.0 | 86.7 | <.001^a^ | 79.7 | 48.0 | <.001^b^ | 95.9 | 71.3 | <.001^b^ |
| Yes | 160 (7.9) | 1.4 | 0.9 |  | 5.0 | 13.3 |  | 20.3 | 52.0 |  | 4.1 | 28.7 |  |
| **Residential areas, %** |  |  |  |  |  |  |  |  |  |  |  |  |  |
| Urban | 1159 (57.0) | 55.0 | 35.7 | <.001^b^ | 63.3 | 60.3 | .422^b^ | 63.8 | 78.3 | .023^a^ | 59.1 | 53.0 | .008^b^ |
| Rural | 874 (43.0) | 45.0 | 64.3 |  | 36.7 | 39.7 |  | 36.2 | 21.7 |  | 40.9 | 47.0 |  |
| **MoCA-J score at the baseline,** Mean±SD | 22.30±3.97 | 23.97±  3.10 | 22.34±  3.38 | <.001 | 22.36±  3.53 | 20.96±  4.20 | <.001 | 19.75±  3.76 | 17.10±  4.71 | <.001 | 23.04±  3.50 | 20.84±  4.41 | <.001 |
| **MoCA-J score at the follow-up,** Mean±SD | 23.03±3.90 | 24.03±  3.49 | - | - | 22.33±  3.87 | - | - | 19.38±  4.54 | - | - | 23.03±  3.90 | - | - |

Abbreviation: LTC, long-term care; MoCA-J, the Japanese version of the Montreal Cognitive Assessment; SD, Standard Deviation.

^a^ *P*-values from Person’s Chi-square test. ^b^ *P*-values from Fisher’s exact test for categorical variables and independent t-test for continuous variable.
