# Supplementary material for: The importance of stroke as a risk factor of cognitive decline in community dwelling older and oldest peoples: the SONIC study
Source: BMC Geriatr. 2020 Jan 22;20:24. doi: 10.1186/s12877-020-1423-5 (PMC6977260; doi:10.1186/s12877-020-1423-5)
Supplement: Supplementary file 4 — Additional file 4: Table S4. Comparison of stroke and non-stroke ratio between follow-up and dropped-out groups. [file 12877_2020_1423_MOESM4_ESM.doc]

**Additional file 4: Table S4.** Comparison of stroke and non-stroke ratio between follow-up and dropped-out groups

| **Ages** | **Stroke group**  **Follow-up : Dropped-out** | **Non-stroke group**  **Follow-up : Dropped-out** | **Follow-up**  **Stroke : Non-stroke** | **Dropped-out**  **Stroke : Non-stroke** |
| --- | --- | --- | --- | --- |
| 70 years old | 1 : 0.39 | 1 : 0.49 | 1 : 19.45 | 1 : 24 |
| 80 years old | 1 : 0.94 | 1 : 0.63 | 1 : 16.85 | 1 : 11.39 |
| 90 years old | 1 : 4.67 | 1 : 2.78 | 1 : 10.5 | 1 : 6.25 |
| All ages | 1 : 1 | 1 : 0.67 | 1 : 17.51 | 1 : 11.67 |
