# Supplementary material for: The importance of stroke as a risk factor of cognitive decline in community dwelling older and oldest peoples: the SONIC study
Source: BMC Geriatr. 2020 Jan 22;20:24. doi: 10.1186/s12877-020-1423-5 (PMC6977260; doi:10.1186/s12877-020-1423-5)
Supplement: Supplementary file 5 — Additional file 5: Table S5. Comparison of baseline characteristics between those with maintained and declined MoCA-J scores between age < 80 years old and age ≥ 80 years old (n = 1333). [file 12877_2020_1423_MOESM5_ESM.docx]

**Additional file 5: Table S5.** Comparison of baseline characteristics between those with maintained and declined MoCA-J scores between age < 80 years old and age ≥ 80 years old (n=1,333)

| **Characteristics** | **Maintained MoCA-J** | | | | **Declined MoCA-J** | | | |
| --- | --- | --- | --- | --- | --- | --- | --- | --- |
|  | **Total**  **n (%)** | **Age < 80**  **years old**  **n=508 (52.6 %)** | **Age ≥ 80**  **years old**  **n=457 (47.4 %)** | ***P*-value** | **Total**  **n (%)** | **Age < 80**  **years old**  **n=167 (45.4 %)** | **Age ≥ 80**  **years old**  **n=201 (54.6 %)** | ***P*-value** |
| **Sex,** % |  |  |  |  |  |  |  |  |
| Male | 482 (49.9) | 48.6 | 51.4 | .402^b^ | 175 (47.6) | 46.7 | 48.3 | .834^b^ |
| Female | 483 (50.1) | 51.4 | 48.6 |  | 193 (52.4) | 53.3 | 51.7 |  |
| **Hypertension,** % |  |  |  |  |  |  |  |  |
| No | 248 (25.7) | 34.1 | 17.4 | <.001^b^ | 95 (25.8) | 37.0 | 17.4 | <.001^b^ |
| Yes | 701 (72.6) | 65.9 | 82.6 |  | 268 (72.8) | 63.0 | 82.6 |  |
| **Diabetes mellitus,** % |  |  |  |  |  |  |  |  |
| No | 773 (80.1) | 86.2 | 85.3 | .704^b^ | 281 (76.4) | 77.7 | 86.0 | .062^b^ |
| Yes | 128 (13.3) | 13.8 | 14.7 |  | 60 (16.3) | 22.3 | 14.0 |  |
| **Dyslipidemia,** % |  |  |  |  |  |  |  |  |
| No | 356 (36.9) | 36.4 | 39.0 | .421^b^ | 148 (40.2) | 41.9 | 41.5 | 1.000^b^ |
| Yes | 589 (61.0) | 63.6 | 61.0 |  | 207 (56.3) | 58.1 | 58.5 |  |
| **Atrial fibrillation,** % |  |  |  |  |  |  |  |  |
| No | 944 (97.8) | 98.4 | 97.2 | .177^a^ | 360 (97.8) | 98.2 | 97.5 | .651^a^ |
| Yes | 21 (2.2) | 1.6 | 2.8 |  | 8 (2.2) | 1.8 | 2.5 |  |
| **Current smoking,** % |  |  |  |  |  |  |  |  |
| No | 847 (87.8) | 82.9 | 96.4 | <.001^b^ | 318 (86.4) | 84.8 | 91.3 | .058^a^ |
| Yes | 102 (10.6) | 17.1 | 3.6 |  | 42 (11.4) | 15.2 | 8.7 |  |
| **Educational level,** % |  |  |  |  |  |  |  |  |
| < 10 years | 249 (25.8) | 24.8 | 27.0 | .289^a^ | 105 (28.5) | 23.8 | 32.8 | .163^a^ |
| 10-12 years | 406 (42.1) | 44.5 | 39.5 |  | 159 (43.2) | 46.3 | 41.3 |  |
| > 12 years | 309 (32.0) | 30.7 | 33.6 |  | 101 (27.4) | 29.9 | 25.9 |  |
| **Frequency of going outdoors,** % |  |  |  |  |  |  |  |  |
| < 1 time/week | 59 (6.1) | 4.7 | 7.7 | <.001^a^ | 20 (5.4) | 3.0 | 7.5 | .092^a^ |
| 1-2 times/week | 120 (12.4) | 8.7 | 16.7 |  | 49 (13.3) | 10.9 | 15.4 |  |
| 3 or 4 times/week | 196 (20.3) | 17.6 | 23.5 |  | 79 (21.5) | 19.4 | 23.4 |  |
| 5 or 6 times/week | 191 (19.8) | 21.3 | 18.2 |  | 80 (21.7) | 23.6 | 20.4 |  |
| Every day | 397 (41.1) | 47.7 | 34.0 |  | 138 (37.5) | 43.0 | 33.3 |  |
| **LTC service used,** % |  |  |  |  |  |  |  |  |
| No | 880 (91.2) | 98.7 | 93.1 | <.001^a^ | 340 (92.4) | 98.1 | 94.0 | .056^a^ |
| Yes | 37 (3.8) | 1.3 | 6.9 |  | 15 (4.1) | 1.9 | 6.0 |  |
| **Residential areas,** % |  |  |  |  |  |  |  |  |
| Urban | 569 (59.0) | 53.9 | 64.6 | .001^b^ | 219 (59.5) | 58.1 | 60.7 | .670^b^ |
| Rural | 396 (41.0) | 46.1 | 35.4 |  | 149 (40.5) | 41.9 | 39.3 |  |
| **MoCA-J score at the baseline,**  Mean±SD | 22.55±3.43 | 23.49±3.06 | 21.49±3.52 | <.001 | 24.33±3.36 | 25.43±2.75 | 23.42±3.56 | <.001 |
| **MoCA-J score at the follow-up,**  Mean±SD | 23.99±3.32 | 24.84±2.79 | 23.06±3.60 | <.001 | 20.51±4.19 | 21.55±4.17 | 19.65±4.01 | <.001 |

Abbreviation: LTC, long-term care; MoCA-J, the Japanese version of the Montreal Cognitive Assessment; SD, Standard Deviation.

^a^ *P*-values from Person’s Chi-square test. ^b^ *P*-values from Fisher’s exact test for categorical variables and independent t-test for continuous variable.
