# Supplementary material for: The importance of stroke as a risk factor of cognitive decline in community dwelling older and oldest peoples: the SONIC study
Source: BMC Geriatr. 2020 Jan 22;20:24. doi: 10.1186/s12877-020-1423-5 (PMC6977260; doi:10.1186/s12877-020-1423-5)
Supplement: Supplementary file 6 — Additional file 6: Table S6. Comparison of baseline characteristics between stroke and non-stroke groups in age < 80 years old and age ≥ 80 years old (n = 1333). [file 12877_2020_1423_MOESM6_ESM.doc]

**Additional file 6: Table S6.** Comparison of baseline characteristics between stroke and non-stroke groups in age < 80 years old and age ≥ 80 years old (n=1,333)

| **Characteristics** | **Stroke** | | | | **Non-stroke** | | | |
| --- | --- | --- | --- | --- | --- | --- | --- | --- |
|  | **Total**  **n (%)** | **Age < 80**  **years old**  **n=33 (45.8 %)** | **Age ≥ 80**  **years old**  **n=39 (54.2 %)** | ***P*-value** | **Total**  **n (%)** | **Age < 80**  **years old**  **n=642 (50.9 %)** | **Age ≥ 80**  **years old**  **n=619 (49.1 %)** | ***P*-value** |
| **Sex,** % |  |  |  |  |  |  |  |  |
| Male | 43 (59.7) | 54.5 | 64.1 | .410^a^ | 614 (48.7) | 47.8 | 49.6 | .536^b^ |
| Female | 29 (40.3) | 45.5 | 35.9 |  | 647 (51.3) | 52.2 | 50.4 |  |
| **Hypertension,** % |  |  |  |  |  |  |  |  |
| No | 9 (12.5) | 19.4 | 7.7 | .148^a^ | 334 (26.5) | 35.6 | 18.0 | <.001^b^ |
| Yes | 61 (84.7) | 80.6 | 92.3 |  | 908 (72.0) | 64.4 | 82.0 |  |
| **Diabetes mellitus,** % |  |  |  |  |  |  |  |  |
| No | 50 (69.4) | 73.3 | 77.8 | .675^a^ | 1004 (79.6) | 84.7 | 86.0 | .564^b^ |
| Yes | 16 (22.2) | 26.7 | 22.2 |  | 172 (13.6) | 15.3 | 14.0 |  |
| **Dyslipidemia,** % |  |  |  |  |  |  |  |  |
| No | 22 (30.6) | 26.7 | 35.9 | .415^a^ | 482 (38.2) | 38.3 | 40.0 | .559^b^ |
| Yes | 47 (65.3) | 73.3 | 64.1 |  | 749 (59.4) | 61.7 | 60.0 |  |
| **Atrial fibrillation,** % |  |  |  |  |  |  |  |  |
| No | 67 (93.1) | 97.0 | 89.7 | .229^a^ | 1237 (98.1) | 98.4 | 97.7 | .360^a^ |
| Yes | 5 (6.9) | 3.0 | 10.3 |  | 24 (1.9) | 1.6 | 2.3 |  |
| **Current smoking,** % |  |  |  |  |  |  |  |  |
| No | 67 (93.1) | 93.9 | 94.7 | .884^a^ | 1098 (87.1) | 82.9 | 94.9 | <.001^b^ |
| Yes | 4 (5.6) | 6.1 | 5.3 |  | 140 (11.1) | 17.1 | 5.1 |  |
| **Educational level,** % |  |  |  |  |  |  |  |  |
| < 10 years | 17 (23.6) | 31.2 | 17.9 | .413^a^ | 337 (26.7) | 24.4 | 29.4 | .056^a^ |
| 10-12 years | 33 (45.8) | 40.6 | 51.3 |  | 532 (42.2) | 45.2 | 39.3 |  |
| > 12 years | 21 (29.2) | 28.1 | 30.8 |  | 389 (30.8) | 30.6 | 31.2 |  |
| **Frequency of going outdoors,** % |  |  |  |  |  |  |  |  |
| < 1 time/week | 9 (12.5) | 12.1 | 12.8 | .638^a^ | 70 (5.6) | 3.9 | 7.3 | <.001^a^ |
| 1-2 times/week | 13 (18.1) | 15.2 | 20.5 |  | 156 (12.4) | 8.9 | 16.0 |  |
| 3 or 4 times/week | 15 (20.8) | 18.2 | 23.1 |  | 260 (20.6) | 18.0 | 23.5 |  |
| 5 or 6 times/week | 9 (12.5) | 9.1 | 15.4 |  | 262 (20.8) | 22.5 | 19.1 |  |
| Every day | 26 (36.1) | 45.5 | 28.2 |  | 509 (40.4) | 46.6 | 34.1 |  |
| **LTC service used,** % |  |  |  |  |  |  |  |  |
| No | 63 (87.5) | 100.0 | 84.6 | .025^a^ | 1157 (91.8) | 98.5 | 93.9 | <.001^b^ |
| Yes | 6 (8.3) | 0.0 | 15.4 |  | 46 (3.6) | 1.5 | 6.1 |  |
| **Residential areas** |  |  |  |  |  |  |  |  |
| Urban | 41 (56.9) | 51.5 | 61.5 | .392^a^ | 747 (59.2) | 55.1 | 63.5 | .003^b^ |
| Rural | 31 (43.1) | 48.5 | 38.5 |  | 514 (40.8) | 44.9 | 36.5 |  |
| **MoCA-J score at the baseline,**  Mean±SD | 22.57±3.91 | 23.64±3.57 | 21.67±4.01 | .032 | 23.07±3.48 | 23.99±3.07 | 22.11±3.62 | <.001 |
| **MoCA-J score at the follow-up,**  Mean±SD | 22.12±4.94 | 23.06±5.27 | 21.33±4.56 | .140 | 23.09±3.83 | 24.07±3.37 | 22.06±4.01 | <.001 |

Abbreviation: LTC, long-term care; MoCA-J, the Japanese version of the Montreal Cognitive Assessment; SD, Standard Deviation.

^a^ *P*-values from Person’s Chi-square test. ^b^ *P*-values from Fisher’s exact test for categorical variables and independent t-test for continuous variable.
