# Supplementary material for: The importance of stroke as a risk factor of cognitive decline in community dwelling older and oldest peoples: the SONIC study
Source: BMC Geriatr. 2020 Jan 22;20:24. doi: 10.1186/s12877-020-1423-5 (PMC6977260; doi:10.1186/s12877-020-1423-5)
Supplement: Supplementary file 7 — Additional file 7: Table S7. Statin used in both stroke/non-stroke and maintained/declined groups (n = 1333). [file 12877_2020_1423_MOESM7_ESM.doc]

**Additional file 7: Table S7** Statin used in both stroke/non-stroke and maintained/declined groups (n=1,333)

| **Stroke experiences** | **MoCA-J status** | **Using Statin** | | **p-value** | | |
| --- | --- | --- | --- | --- | --- | --- |
|  |  | **Yes** | **No** | **Stroke experiences vs. MoCA-J status** | **Stroke experiences**  **(yes vs. no)** | **MoCA-J status**  **(maintained vs. declined)** |
| Yes | Maintained MoCA-J  Declined MoCA-J | 10 (22.7)  10 (35.7) | 34 (77.3)  18 (64.3) | .230^a^ | .244^a^ | .555^a^ |
| No | Maintained MoCA-J  Declined MoCA-J | 209 (22.7)  67 (19.8) | 712 (77.3)  272 (80.2) | .283^b^ |  |  |

Abbreviation: MoCA-J, the Japanese version of the Montreal Cognitive Assessment.

^a^ *P*-values from Person’s Chi-square test. ^b^ *P*-values from Fisher’s exact test for categorical variables and independent t-test for continuous variable.
